# New Appliances and Things Medical

**Published:** 1907-07-06

**Authors:** 


### NEW APPLIANCES AND THINGS MEDICAL

### BAYER'S PHARMACEUTICAL PRODUCTS.

(The Bayer Company, Ltd., 19 St. Dunstan's Hill, London, E.C.)

We have received from this firm samples of their compressed tablets of the following preparations :—Tannigen (5 grains), Helmitol (5 grains), Trional Bayer (5 grains), Aspirin (5 grains), Veronal (5 grains), and Heroin hydrochloride ($\frac{1}{24}$ grain). These tablets consist of the pure drug, compressed and uncoated. They are extremely convenient for administration, and should prove of great service to the practitioner who desires to try any of the more modern drugs which the synthetic chemist has placed on the market.

